# Five genomes, same suspects: a comparative filter for plant GWAS candidates

**DOI:** 10.1093/plphys/kiag329

**Published:** 2026-06-03

**Authors:** Neeta Lohani

**Affiliations:** Assistant Features Editor, Plant Physiology, American Society of Plant Biologists; Department of Biotechnology, Thapar Institute of Engineering and Technology, Bhadson Rd, Prem Nagar, Patiala, Punjab 147004, India

Plant genome-wide association studies (GWAS) reliably point to where a trait is encoded in the genome, but pinpointing which gene at that region actually determines the phenotype has been proven far more difficult. Solving the genotyping side of the problem has progressed rapidly. Marker densities have climbed from a few thousand single-nucleotide polymorphisms (SNPs) to whole-genome resequencing of hundreds of accessions. GWAS is now routinely applied across most major crop and model species, recovering robust marker–trait associations for nearly every quantitative trait of interest (Liu and Yan 2019; Alqudah et al. 2020). What has not kept pace is the ability to move from markers to gene. Linkage disequilibrium (LD) spreads each association signal across a block of dozens to thousands of physically close genes (Cubillos, Coustham and Loudet 2012), and no amount of statistical refinement can reliably single out the functional variant. To narrow these lists, researchers turn to prior biological knowledge, ranking candidates by ontology terms, expression patterns, or membership in curated gene lists. These strategies are effective when the causal gene is already well characterized, but they offer little guidance when it is not. This creates a well-documented feedback loop in which the same familiar genes attract a disproportionate share of follow-up effort, while thousands of equally plausible candidates go untested for decades (Stoeger et al. 2018; Baxter 2020). The trait-to-gene gap, therefore, continues to limit how much biology we can extract from each new GWAS dataset.

Ionomic traits describe the concentrations of minerals and trace elements that plants accumulate in their tissues. These traits have one useful property for the trait-to-gene problem. Elements are universal phenotypes: iron is iron whether measured in *Arabidopsis* seeds or maize kernels. The machinery that takes up, transports, and stores these elements is also largely conserved across angiosperms. Several well-studied cases support this point. Natural variation at the *HKT1* sodium transporter shapes sodium accumulation in both Arabidopsis and rice (Rus et al. 2006). The *MOT1* molybdate transporter controls molybdenum accumulation across distantly related species (Baxter et al. 2008). The *HMA3* cadmium pump determines leaf cadmium in Arabidopsis and grain cadmium in rice (Chao et al. 2012). These examples raise a practical question. Can the recurrence of orthologs at GWAS loci across species be used as a formal statistical filter for finding conserved trait drivers?

In a recent article published in *Plant Physiology*, Whitt et al. (2026) answered this question by introducing FiReMAGE (Filtering Results of Multi-species, Analogous, GWAS Experiments). The pipeline is open source and available at https://github.com/danforthcenter/FiReMAGE. The authors applied FiReMAGE (Fig. 1A) to published ionomic GWAS for 19 elements measured in seeds of 5 flowering plants: *Arabidopsis thaliana*, soybean (*Glycine max*), rice (*Oryza sativa*), sorghum (*Sorghum bicolor*), and maize (*Zea mays*). For each species, the authors first collapsed significant SNPs into loci using species-specific LD ranges. They then extracted every gene falling within each locus. Finally, they linked genes across species using OrthoFinder-derived orthogroups (Emms and Kelly 2019). The number of loci differed widely between species. Rice contributed a few hundred loci, whereas soybean contributed several thousand. These differences reflect variation in population size, marker density, and LD extent. Orthogroups that appeared at the same trait in 2 or more species were retained as conserved candidates.

**Figure 1:**
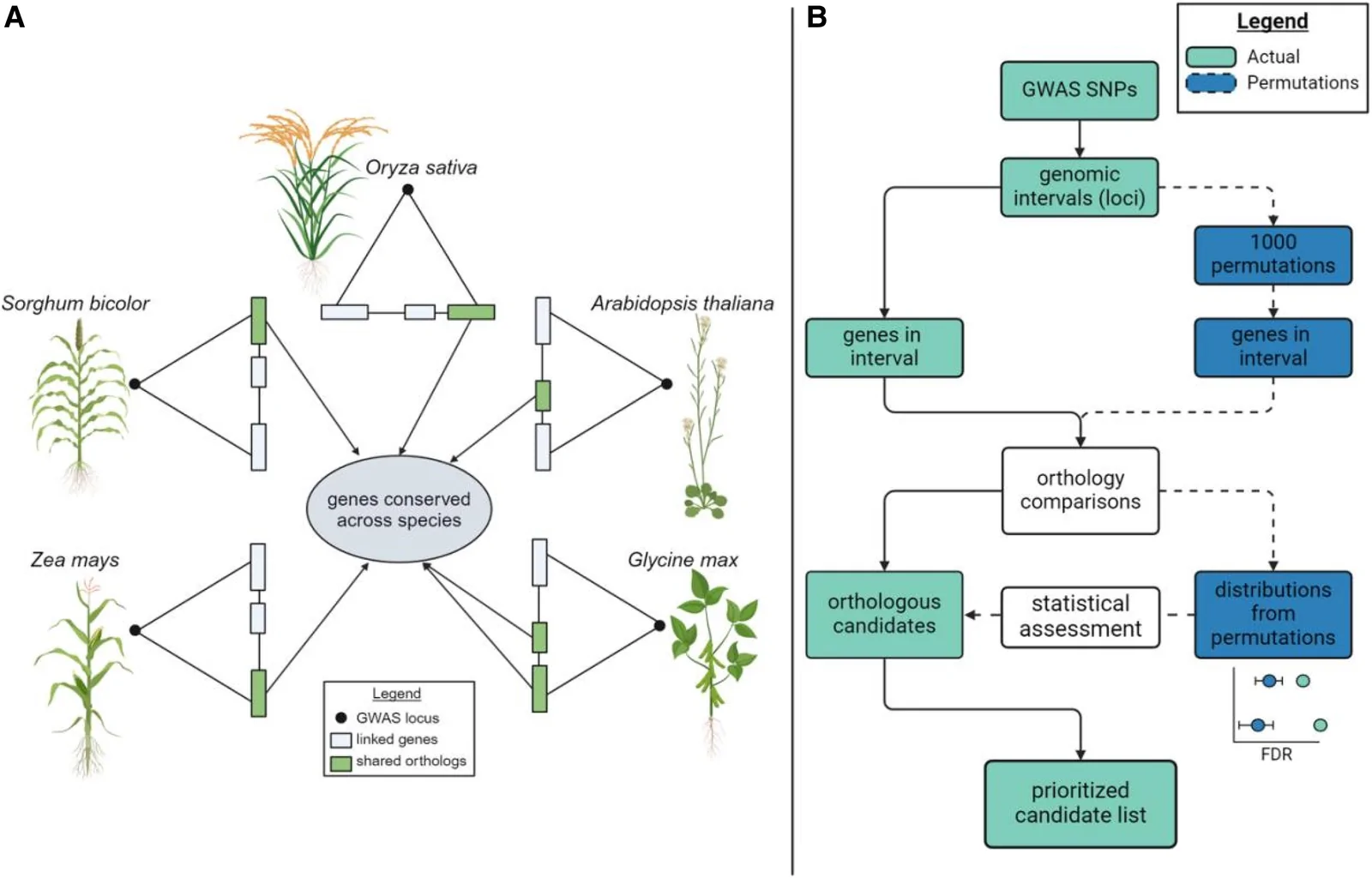
Overview of the FiReMAGE pipeline. (A) Across 5 flowering plant species (*Sorghum bicolor*, *Oryza sativa*, *Arabidopsis thaliana*, *Zea mays*, and *Glycine max*), genes within ionomic GWAS loci (white boxes) are linked through OrthoFinder-derived orthogroups, and orthologs shared between species (green boxes) converge into a set of genes conserved across species. (B) Statistical workflow: GWAS SNPs are collapsed into genomic intervals, and the resulting genes (green, actual) are compared in parallel with those from 1,000 size-matched random permutations (blue) to generate an empirical null. A false discovery rate (FDR) derived from this null prioritizes the orthologous candidate list. Adapted from Whitt et al. (2026).

The authors built a permutation framework to separate genuine cross-species recurrence from chance (Fig. 1B). For each species, the authors generated 1,000 sets of randomly placed genomic intervals. Each set was matched in number and size to the real GWAS loci of that species. These randomized intervals were then passed through the same orthology pipeline used for the real data. The observed number of recurrent orthogroups was compared to this empirical null to derive a false discovery rate (FDR) for every species–trait–composition combination. The permutation preserves locus size, marker density, and LD structure for each dataset. As a result, the framework directly handles methodological heterogeneity that has long frustrated cross-species GWAS comparisons. Sorghum and maize share substantial synteny, which could in principle inflate the conservation signal. The authors checked this directly. FDRs for orthogroups containing the sorghum–maize pair were not elevated, showing that the permutation control accounts for shared chromosomal organization.

FiReMAGE recovered classical regulators of elemental accumulation at the expected trait–element pairings. These included *MOT1* for molybdenum, *HMA3* for cadmium, and *BTS*, *NRAMP3*, *MTP11*, and *PHT1;4* for their respective elements. Across all 19 traits, the curated Known Ionomic Gene (KIG) list (Whitt et al. 2020) was significantly enriched in real GWAS loci compared with permuted regions, confirming the underlying orthology signal. The bigger gain came from candidate coverage. The extended KIG list intersected fewer than 5% of GWAS loci across the 5 species. In contrast, FiReMAGE returned a conserved candidate for over 30% of loci in soybean and maize, and for more than 50% of loci in Arabidopsis, rice, and sorghum. This is an order-of-magnitude expansion in the proportion of GWAS signals that come with a high-value gene call. Across the 19 elements, 81 candidate genes distributed across 9 orthogroups were recovered in all 5 species. The top-ranked candidates by permutation-derived FDR were also enriched for the most significant SNP–trait associations in the original GWAS.

Next, the authors examined the conserved candidates that lie outside the curated list. Members of a Domain of Unknown Function 567 (DUF567) family were recovered in all 5 species. This family is root-expressed in Arabidopsis and has previously been linked to metal hyperaccumulation in *Arabidopsis halleri* (Filatov et al. 2006). RAB GTPase homologs implicated in cell-wall composition were also recovered across the 5 species. These are precisely the kinds of leads that annotation-based prioritization tends to overlook, because they lack preexisting functional evidence. FiReMAGE also rescued genes that would not survive a single-species significance threshold. *MOT2* in soybean and members of the *AtHMA2/3/4* tandem array in Arabidopsis are 2 such cases. Both have since been confirmed as drivers of ionomic variation (Chao et al. 2012). FiReMAGE therefore confirms known regulators and surfaces well-supported new candidates that would otherwise be dismissed as statistical noise.

FiReMAGE has clear boundaries. By construction, the pipeline will miss taxonomically restricted contributions to elemental homeostasis. Glucosinolate-linked sulfur handling in Arabidopsis and nodulation-associated mineral fluxes in legumes are 2 examples. These mechanisms have no orthologs in the other species and therefore fall outside the comparative filter. The overlap between FiReMAGE candidates and those identified by network-guided prioritization in maize (Schaefer et al. 2018) was also minimal. This indicates that comparative orthology and lineage-specific co-expression illuminate different layers of trait architecture. The 2 approaches are complementary rather than redundant. FiReMAGE itself requires only 3 inputs: GWAS intervals, gene coordinates, and an orthology table. All 3 are now available for a growing list of crops. Any conserved quantitative trait, including root architecture, flowering time, or seed composition, can in principle be analyzed through the same framework. As high-throughput phenotyping continues to outpace functional follow-up in plants, comparative filters like FiReMAGE provide a practical route for converting long candidate lists into testable hypotheses. The direct beneficiaries are biofortification, food safety, and crop nutritional quality.

**Recent related articles published in *Plant Physiology***

- Cheng et al. (2024) mapped quantitative trait loci for cadmium accumulation across 12 distinct tissues of a Polish wheat (*Triticum polonicum* L.) recombinant inbred line population. The authors identified 34 major quantitative trait loci, of which 29 were novel. The work also uncovered pleiotropic regulators of Cd uptake, translocation, and grain loading. It offers a complementary, single-species view of the same conserved-trait architecture that FiReMAGE detects across taxa.
- Wang et al. (2025) systematically assessed how genome assemblies, genotyping approaches, variant types, allelic complexities, polyploidy, and population structure shape genomic prediction of 20 complex traits in switchgrass (*Panicum virgatum* L.), an allotetraploid biofuel feedstock. The study provides a parameter-level dissection of the methodological choices that determine the resolution of quantitative genetic discovery in non-model crops.

## Data Availability

No new data were generated or analyzed in support of this article.

## References

[kiag329-B1] Alqudah AM, Sallam A, Stephen Baenziger P, Börner A. 2020. GWAS: fast-forwarding gene identification and characterization in temperate cereals: lessons from barley–a review. J Adv Res. 22:119–135. 10.1016/j.jare.2019.10.013. 31956447 PMC6961222

[kiag329-B2] Baxter I et al 2008. Variation in molybdenum content across broadly distributed populations of Arabidopsis thaliana is controlled by a mitochondrial molybdenum transporter (MOT1). PLoS Genet. 4:e1000004. 10.1371/journal.pgen.1000004. 18454190 PMC2265440

[kiag329-B3] Baxter I . 2020. We aren’t good at picking candidate genes, and it's slowing us down. Curr Opin Plant Biol. 54:57–60. 10.1016/j.pbi.2020.01.006. 32106014

[kiag329-B4] Chao D-Y et al 2012. Genome-wide association studies identify heavy metal ATPase3 as the primary determinant of natural variation in leaf cadmium in Arabidopsis thaliana. PLoS Genet. 8:e1002923. 10.1371/journal.pgen.1002923. 22969436 PMC3435251

[kiag329-B5] Cheng Y et al 2024. Genetic factors of grain cadmium concentration in Polish wheat (Triticum polonicum L.). Plant Physiol. 196:979–995. 10.1093/plphys/kiae353. 38917222

[kiag329-B6] Cubillos FA, Coustham V, Loudet O. 2012. Lessons from eQTL mapping studies: non-coding regions and their role behind natural phenotypic variation in plants. Curr Opin Plant Biol. 15:192–198. 10.1016/j.pbi.2012.01.005. 22265229

[kiag329-B7] Emms DM, Kelly S. 2019. OrthoFinder: phylogenetic orthology inference for comparative genomics. Genome Biol. 20:238. 10.1186/s13059-019-1832-y. 31727128 PMC6857279

[kiag329-B8] Filatov V et al 2006. Comparison of gene expression in segregating families identifies genes and genomic regions involved in a novel adaptation, zinc hyperaccumulation. Mol Ecol. 15:3045–3059. 10.1111/j.1365-294X.2006.02981.x. 16911220

[kiag329-B9] Liu HJ, Yan J. 2019. Crop genome-wide association study: a harvest of biological relevance. Plant J. 97:8–18. 10.1111/tpj.14139. 30368955

[kiag329-B10] Rus A et al 2006. Natural variants of at HKT1 enhance Na+ accumulation in two wild populations of Arabidopsis. PLoS Genet. 2:e210. 10.1371/journal.pgen.0020210. 17140289 PMC1665649

[kiag329-B11] Schaefer RJ et al 2018. Integrating coexpression networks with GWAS to prioritize causal genes in maize. Plant Cell. 30:2922–2942. 10.1105/tpc.18.00299. 30413654 PMC6354270

[kiag329-B12] Stoeger T, Gerlach M, Morimoto RI, Nunes Amaral LA. 2018. Large-scale investigation of the reasons why potentially important genes are ignored. PLoS Biol. 16:e2006643. 10.1371/journal.pbio.2006643. 30226837 PMC6143198

[kiag329-B13] Wang P et al 2025. Optimizing genomic prediction for complex traits via investigating multiple factors in switchgrass. Plant Physiol. 198:kiaf188. 10.1093/plphys/kiaf188. 40331363 PMC12238539

[kiag329-B14] Whitt L et al 2020. A curated list of genes that affect the plant ionome. Plant Direct. 4:e00272. 10.1002/pld3.272. 33103043 PMC7576880

[kiag329-B15] Whitt L et al 2026. A comparative approach for selecting orthologous candidate genes in GWAS across multiple species. Plant Physiol. 201:kiag298. 10.1093/plphys/kiag298. PMC13353110 42184341

